# Smaller Anterior Cruciate Ligament Diameter Is a Predictor of Subjects Prone to Ligament Injuries: An Ultrasound Study

**DOI:** 10.1155/2015/845689

**Published:** 2015-01-22

**Authors:** Parag Suresh Mahajan, Prem Chandra, Vidya Chander Negi, Abhilash Pullincherry Jayaram, Sheik Akbar Hussein

**Affiliations:** ^1^Department of Clinical Imaging, Hamad Medical Corporation, P.O. Box 3050, Doha, Qatar; ^2^Medical Research Center, Hamad Medical Corporation, P.O. Box 3050, Doha, Qatar

## Abstract

*Purpose*. To test if diameter of normal anterior cruciate ligament (ACL) can be measured by ultrasound (US), to see if there is a relationship between smaller ACL diameter and ACL injury, and to assess agreement between radiologists in measuring ACL diameter in cases and matched controls. *Materials and Methods*. In this ethics committee-approved study, maximum diameter of ACL near tibial insertion site was measured by static and dynamic US study in 25 normal contralateral knees of subjects who suffered noncontact ACL injury and in 25 matched control subjects. *Results*. ACL was visualized as a thick linear hypoechoic band inserted approximately 11 mm caudal to the tibial plateau and the intercondylar eminence. Maximum diameter of contralateral ACL near tibial insertion site among injured subjects was significantly smaller than in noninjured subjects (0.62 ± 0.07 cm versus 0.81 ± 0.06 cm; *P* < 0.0001). In the regression analysis, the diameter of ACL near tibial insertion site was found significantly proportional to body weight and not significantly associated to height, gender, and age. *Conclusion*. Diameter of normal ACL near tibial insertion site can be measured by US and the maximum diameter is significantly smaller among subjects with noncontact ACL injury. US is a promising modality that can be used as an excellent screening test to detect subjects especially aspiring athletes prone to ACL injury. Very strong agreement was observed between radiologists in measuring ACL diameter.

## 1. Background

ACL is one of the important ligaments of knee. It is entirely intracapsular and lies in the distal femoral intercondylar notch. It extends caudally from posteromedial surface of the lateral condyle of femur to the anterior part of the proximal tibial intercondylar area. ACL is also the most commonly injured knee ligament in athletes and can cause significant morbidity in all age groups. Although numerous procedures have been devised to treat ACL tears, they have limited cure rates. Furthermore chronically altered mechanical properties of knee secondary to ACL injury may cause complications like chondromalacia and osteoarthritis [1–3]. ACL injuries can thus be particularly devastating to young athletes. Therefore, it is essential to find techniques or processes to foresee the risk of injury to ACL to adopt appropriate preventive measures.

Approximately three-fourths of mechanical injuries of ACL are noncontact type, suggesting that early recognition of this risk may help in the prevention of injury [3]. It has been demonstrated that the relative risk of a noncontact ACL injury is almost 10 times more in female sportspersons from different colleges than their male counterparts [4]. Significant research is being done to ascertain constitutional and physical properties of the ACL. Relationship between volume and cross-sectional area (CSA) of ACL and body weight, height, age, and gender has been established. ACL in females is inferior in strength and smaller in size than males, which is additionally proven to be unrelated to height [5–8]. This suggests that a narrower ACL may be a predisposing factor for noncontact injury of ACL.

Since physical attributes of every single fibril constituting the ACL and other ligaments of a person are identical [9] and mean fibril diameter is uniform across sexes [6], a thinner ACL may just have less number of fibrils, resulting in inferior strength. Diameter, CSA, and even the volume can appropriately and adequately describe the size of the ACL, although volume is a more suitable* in vivo* measurement than a uniplanar measurement of CSA or diameter. A recent study reports that MRI measured smaller ACL volume correlates with ACL injury when weight, height, gender, and age are kept almost constant [10]. However it is debatable whether performing an MRI of the knee in an athlete without symptoms is justifiable [11]. Surprisingly there are a very few studies directly or indirectly examining the ACL by ultrasound despite it being an ideal (wide availability, quick completion, and economical and nonionizing radiation nature) screening examination to assess the size of a normal ACL [12–15]. Hence this study was designed to

1. evaluate usefulness of ultrasound in measuring diameter of normal ACL;
2. test if there exists a relationship between smaller ACL diameter and ACL injury, by measuring the ACL diameter in contralateral knees of ACL injured subjects and comparing them to ACL size in weight, height, gender, and age matched controls;
3. assess agreement between two radiologists in measuring ACL diameter in cases and matched controls.

## 2. Materials and Methods

### 2.1. Subject Population

First 25 subjects undergoing knee MRI at our hospital who satisfied the following inclusion criteria were prospectively studied:

- subjects in whom ACL injury has been diagnosed on MRI;
- being a self-reported noncontact ACL injury;
- those who are willing to participate in the study;
- those who sign our institutional review board-approved informed consent.

The following exclusion criteria were used:

- history of trauma to the soft tissue of the lower extremity requiring surgical repair/reconstruction (excluding ACL tears);
- meniscectomy of greater than one-fourth of the meniscus;
- fracture of lower extremity bones requiring internal fixation.

Also, another 25 control subjects with normal or noninjured ACL who match the above selected subjects in gender, age, weight, height, and ethnicity were invited to participate in the study. Most of the control subjects were selected from amongst the patients who underwent MRI of knee at our hospital and who had MRI-confirmed normal/noninjured ACL. Static and dynamic ultrasound study of the normal knees of all 50 subjects was performed to measure maximum diameter of ACL near tibial insertion site.

The sonographer was blinded as to the status of the subject being examined. Agreement between the sonographers was studied. In addition to the main objectives, correlation between diameter of ACL and weight, height, and age of the subject was also studied. We did one to one matching between the injured subjects and the matched controls for height, weight, gender, and ethnicity and achieved similar mean between groups for age. Each injured subject and the matched control had the same gender and ethnicity. The control subjects were chosen in such a way that the disparity between heights of each and every ACL-injured subject and the matched control was less than 5 centimeters and the disparity between weights of each and every ACL-injured subject and the matched control was less than 3 kilograms. The average difference between the heights of control and injured subjects was within 1 cm (170.7 cm versus 171.2 cm) and the average difference between the weights of control and injured subjects was within 1.5 kg (78.7 kg versus 80.2 kg). No statistically significant disparity was observed between the ACL-injured and control groups for age (*P* = 0.890), height (*P* = 0.768), weight (*P* = 0.685), and gender (*P* = 1.00) based on statistical analysis using an unpaired Student's* t*-test and Chi-square tests (Table 1).

The study was approved by our institutional research ethics committee.

### 2.2. Ultrasound-Based Diameter Calculation

**Figure 1:**
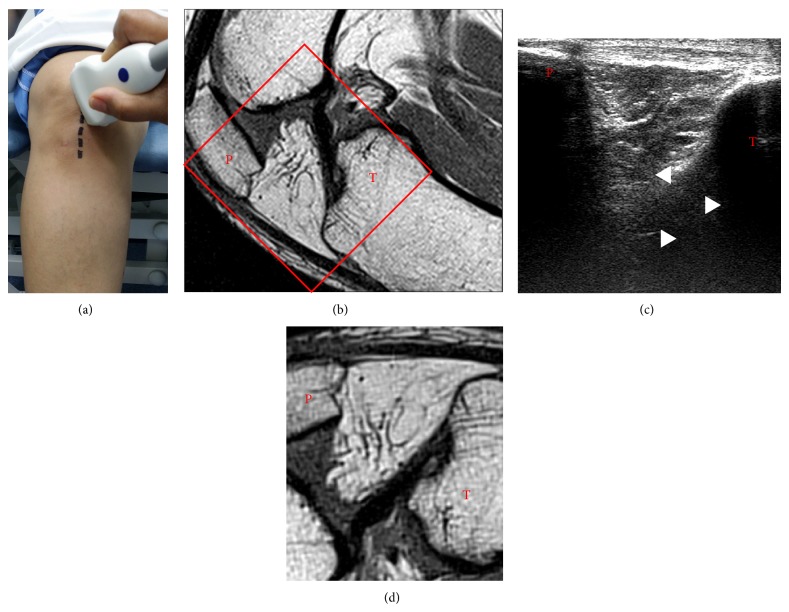
Position of the patient and the ultrasound probe during ultrasound examination of anterior cruciate ligament (ACL) is shown in (a). MRI image of knee in 90-degree flexion is shown in (b). The red rectangle denotes area of the image rotated and presented in (d) to match the morphology as seen in ultrasound image (c). White arrowheads in (c) demonstrate normal ACL. P denotes patella and T denotes tibia.

Only tibial part of ACL could be examined by ultrasound. Maximum diameter of ACL near tibial insertion site was measured by static and dynamic ultrasound (Siemens, Munich, Germany) study using high frequency (7–9 MHz) linear transducer with the subject's knee in 90-degree flexion in supine position. While examining the ACLs, we asked the subjects to internally or externally rotate their leg to perform the dynamic ultrasound examination. The linear high frequency ultrasound probe was placed on the subject's skin inferior to patella such that its long axis is parallel to that of the ACL. This was achieved by rotating the superior part of the probe externally by 30 degrees (Figure 1). The ACL was visualized as a thick linear hypoechoic band like structure inserted approximately 11 mm caudal to the tibial plateau and intercondylar eminence (Figure 1). The maximum diameter of ACL near tibial insertion site was measured for 25 opposite, normal, or unaffected knees of persons who suffered noncontact injury to ACL and for 25 controls matched for weight, height, gender, and age. Each ACL was measured separately by two radiologists who were blinded to the status of the subject (injured or control group). Interobserver variation was calculated. Average of the two measurements was considered for statistical analysis. We applied stepwise multiple regression to the statistical data to assess the disparity in the diameter of ACL between injured and control groups. During this analysis, weight, height, age, and gender were deemed to be potential covariates.

### 2.3. Validation of Ultrasound-Based Diameter Calculation

MRI is considered a gold standard for* in vivo* ACL diameter measurement. We used MRI studies of 10 knees of 10 control subjects for validating the ultrasound-based ACL diameter (near tibial insertion site) measurement performed in this study. MRI examinations were performed using a GE Signa 1.5T system. Sagittal 3D-SPGR MRI images with voxel size of 0.055 × 0.055 × 0.15 cm were obtained. The MRI examination was performed on one randomly chosen knee of each of the control subjects. The subject was placed in supine position with knee in 90-degree flexion to mimic the position during ultrasound study. Maximum anteroposterior diameter of each ACL (near tibial insertion site) was measured by two radiologists independently. Interobserver variation was measured. Average of the two measurements was compared with average of maximum ACL diameter estimated by ultrasound. Tate et al. in 2006 demonstrated that a similar MRI based procedure used to measure muscle size produced an error of only 0.8% [16].

### 2.4. Sample Size

As per our review of literature, there are no such studies available in the literature on diameter of ACL in injured and noninjured subjects by ultrasound. Therefore, there is no formal sample size calculation done for this study. We studied a total of 50 subjects/knees, 25 ACL-injured subjects and 25 noninjured matched controls.

### 2.5. Statistical Analysis

Categorical and continuous data values were expressed as frequency (percentage) and mean ± SD, median, and range as appropriate. Descriptive statistics were used to summarize all demographic, clinical, and radiological features of the examined subjects. The primary outcome variable was ACL diameter between injured and noninjured cases. Associations between two or more qualitative variables were assessed using Chi-square test or Fisher's exact test as appropriate. Quantitative variables means between the two independent groups were analyzed using unpaired* t*-test and Mann-Whitney* U* test as appropriate. We used paired* t*-test to compare and examine the difference in mean MRI and US based ACL diameter measured in 10 subjects within control group. We computed the intraclass correlation coefficient (ICC) and constructed the Bland-Altman plot to assess the interobserver variability between two radiologists measuring ACL diameter values. Relationship between two quantitative variables was examined using Pearson's correlation coefficients. We used stepwise multiple regression to assess the disparity in diameter of ACL between cases and controls while considering height, weight, age, and gender as potential covariates. We also investigated and tested the interaction effects. The results were presented with the associated 95% confidence interval. Pictorial presentations of the key results were made using appropriate statistical graphs. A two-sided *P* value less than 0.05 (<0.05) was deemed to be statistically significant. All statistical analyses were done using statistical package PASW Statistics (version 19.0, SPSS Inc., Chicago, USA).

## 3. Results

No significant disparity was observed between the ACL-injured and control groups for age (*P* = 0.890), height (*P* = 0.768), weight (*P* = 0.685), and gender (*P* = 1.00) using unpaired Student's* t*-test and Chi-square tests (Table 1). Contralateral ACL maximum diameter (near tibial insertion site) for ACL-injured subjects was significantly less than that for noninjured matched control subjects (0.62 ± 0.07 versus 0.81 ± 0.06 cm; *P* < 0.0001).

**Figure 2:**
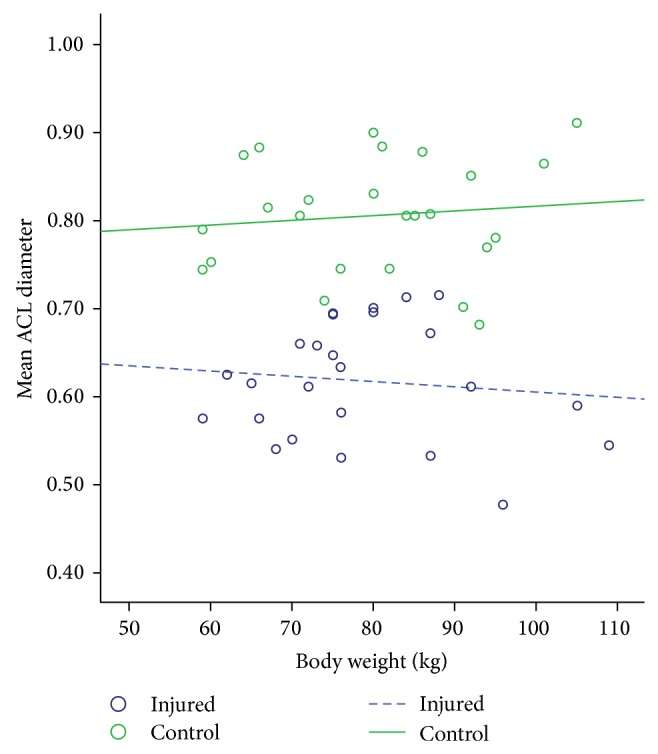
Plot showing mean ACL diameter (cm) in controls and injured cases in relation to body weight (kg).

Stepwise regression analysis showed that body weight was a significant predictor (*R* = 0.357; *P* = 0.016), while height, age, and gender were insignificantly associated with ACL diameter. The mean diameter of the contralateral ACL near tibial insertion site for the injured group was 0.19 cm less than that for a control subject having similar weight (*P* < 0.0001). The 95% confidence interval (CI) of the mean difference was 1.5 to 2.3 (Table 2 and Figure 2). The ACL diameter of all 25 subjects in the ACL-injured group was smaller than their matched controls, and just 6 subjects had lower body weight. For the mean body weight of 78.5 kg for the studied population the ACL-injured group had a mean contralateral ACL maximum diameter (near tibial insertion site) of 0.62 cm, while the control group had a mean diameter (maximum) of 0.81 cm.

**Figure 3:**
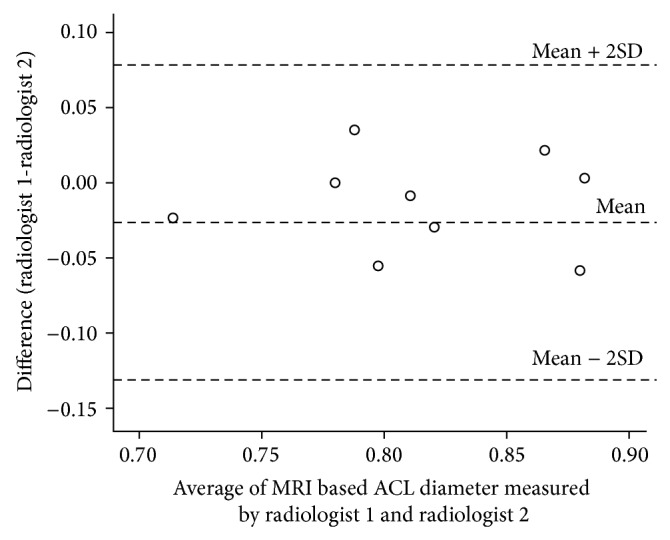
Bland Altman plot: MRI based ACL diameter (cm) measurements in controls by two radiologists.

**Figure 4:**
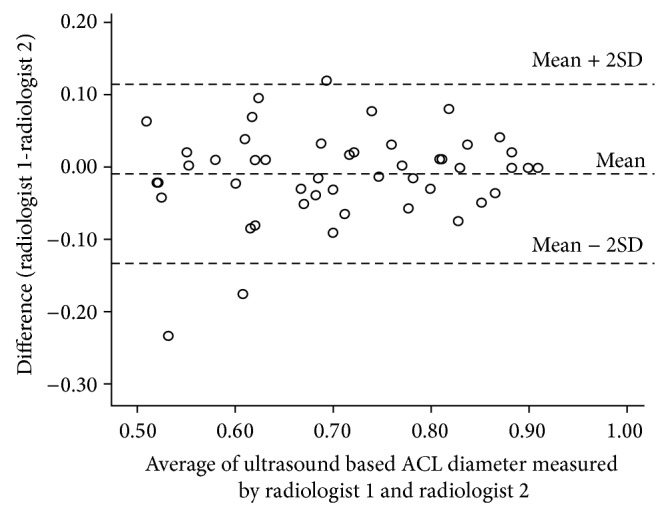
Bland Altman plot: ultrasound based ACL diameter (cm) measurements by two radiologists.

**Figure 5:**
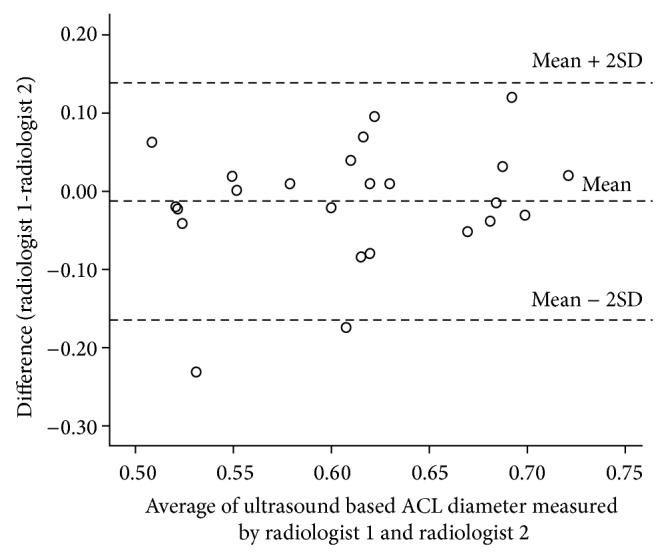
Bland Altman plot: ultrasound based ACL diameter (cm) measurements in injured cases by two radiologists.

**Figure 6:**
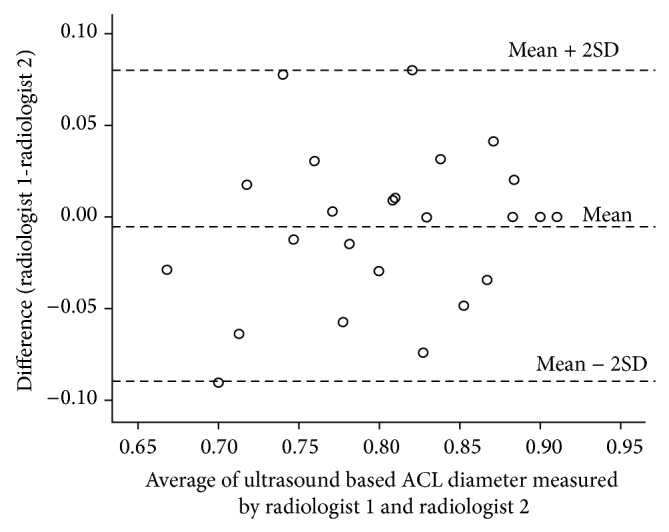
Bland Altman plot: ultrasound based ACL diameter (cm) measurements in controls by two radiologists.

The consensus between the two recurring measures during validation with MRI measurements demonstrated a very strong agreement (intraclass correlation of 0.87; 95% CI: 0.50 to 0.97) (Figure 3). The regression analysis between the MRI-measured diameter and the ultrasound-measured diameter revealed a correlation of 0.070, a slope of 0.052, and a constant offset of 0.041 cm. The regression line was almost similar to *y* = *x* (slope: *P* = 0.05; intercept: *P* = 0.051). We also compared the results of measurements by two radiologists (raters) by conducting an interrater accuracy test. The intraclass correlation coefficient for these 2 radiologists was 0.93 (95% CI: 0.88 to 0.96), indicating a very strong agreement. The limits of Figure 2 show a comparison of ultrasound ACL diameter measured by radiologist 1 and radiologist 2 (Figure 4). Here the mean difference was −0.01 with 95% CI −0.03 to 0.01. Thus radiologist 1 tends to give a lower reading, ranging from −0.03 to 0.01. Despite this, the limits of disagreement are considerably low (high agreement) and hence both radiologists provide similar values measuring ultrasound ACL diameter. Similarly strong agreement was observed when analyzed separately for ACL-injured and control group subjects (Figures 5 and 6). Finally, the investigation of variance showed a mean systematic difference of 0.3 cm between the 2 radiologists.

## 4. Discussion

Maximum diameter of ACL near tibial insertion site was chosen as a criterion for evaluation of ACL size by ultrasound as the ACL is thicker towards tibial insertion and only tibial part of normal ACLs could be consistently examined by ultrasound making it easy to compare with MRI size measurements.

Our study results conclusively demonstrate that subjects who have experienced a noncontact ACL injury have a remarkably smaller diameter of the ACL in their contralateral unaffected knee than matched controls. This in turn concludes that a smaller ACL has less strength thus predisposing it to increased risk of injury. Very few studies have attempted to establish a link between the size of ACL and its predisposition to injury [10, 17]. Our study is one of these and provides a very strong evidence because we directly compared ACL-injured subjects to matched controls. Chandrashekar et al. in 2005 studied ACL volume in males and females and found a remarkable gender difference in ACL volume [18]. They were the first to suggest that ACL size is a significant risk factor for injury. Their suggestion was based on the observed differences in the rates of ACL injury in males and females [18]. Complex interaction and combination of various intrinsic and extrinsic influences lead to significant biomechanical loading that surpasses the failure strength of ACL leading to its rupture [19–21]. Chaudhari et al. in 2009 also directly compared ACL-injured subjects to matched controls and demonstrated a strong link between volume of ACL and ACL injuries [10].

Statistical analysis in this study demonstrated that ACL diameter is significantly proportional to body weight and not significantly associated to height, gender, and age. During statistical analysis if body weight is adjusted, the disparity in the ACL diameter between the ACL-injured and control subjects would be exaggerated [10]. In a similar hypothetical model of regression analysis for knees of two different subjects having identical body weight, the ACL-injured subject would have 0.19 cm smaller contralateral unaffected ACL diameter (near tibial insertion site) than the control subject [10]. This difference in ACL diameter represents approximately 26.6% of the mean ACL diameter (near tibial insertion site) noted in the data and illustrates a significant effect [10, 22].

Screening of athletes to identify potential risk factors predisposing to ACL injury has obvious advantages. Our study identifies diameter (size) of ACL as one such important potential risk factor. Subjects with thinner ACL could negate this risk by undertaking special neuromuscular exercises targeted to decrease the knee loading [9, 23, 24]. Improving the strength of the adjacent muscles and making them function as ACL agonists can significantly decrease the biomechanical loading of ACL, thus decreasing the injury risk [10]. Specific exercise routines that induce ACL hypertrophy may be developed for subjects at risk of ACL injury, especially adolescents. Few molecules inducing proliferation of ACL cells have been identified [10]. More such highly effective molecules may be identified.

Many researchers have studied the relationship between predisposing extrinsic factors like extremity strength, knee joint loading, and neuromuscular control and risk of ACL injury [10, 25–28]. Many researchers have also studied targeted training regimes that can change these extrinsic factors and in turn decrease the ACL injury risk [29, 30]. Other studies have examined the relationship between the predisposing intrinsic factors and ACL injury risk [10, 17]. Screening methodology studying both extrinsic and intrinsic factors may be the best technique that can successfully identify subjects who would benefit most from training regimes targeting injury prevention. Only a handful of studies have extensively examined both extrinsic and intrinsic predisposing factors together, making comparison of relative significance of different factors difficult [15]. More such exhaustive studies are needed as results from only these types of studies can help in deciding appropriateness and cost effectiveness of screening tests at injury risk mitigation.

MRI is more accurate in assessing ACL size than US; however, using MRI of knee to screen a large number of aspiring athletes can be extremely expensive and time consuming and can significantly overburden the already strained healthcare resources. It is thus debatable whether performing an MRI of the knee in an aspiring athlete without symptoms is justifiable [11]. US is a promising modality that can be used as an excellent screening test to detect subjects especially aspiring athletes prone to ACL injury. US can be an ideal screening examination to assess the size of a normal ACL due to its wide availability and short completion time and also due to its economical and nonionizing radiation nature [12–15].

Major limitations of our study include ACL diameter as the measured criteria to determine ACL size and evaluation of only tibial aspect of the ACL by ultrasonography. Since stress in a ligamentous tissue is a product of force divided by cross-sectional area (CSA), minimum CSA would be an excellent measure compared to diameter in assessing ACL size. Measuring cross-sectional area of ACL by ultrasound would require further studies and may be difficult due to inability to clearly separate ACL from adjacent soft tissue. Volume would also be a very good criterion to assess ACL size; however, it is not possible to measure ACL volume by ultrasound. If the ACL is assumed to have a fairly regular and characteristic shape and aspect ratio (CSA/length), the diameter should be an appropriate surrogate measure for CSA [10]. It is possible to examine only the tibial part of ACL by ultrasound and not the whole ACL. Sometimes it may be difficult to distinguish ACL from surrounding tissue on ultrasound. This can be minimized by using dynamic ultrasound. Utilizing the unaffected ACL from opposite extremity to represent the diameter of the torn ACL, which cannot be measured after injury, is also a limitation of the study. However, Jamison et al. in 2009 compared the bilateral knee ACL sizes using MRI in healthy subjects and concluded that no remarkable disparity is present between normal unaffected ACLs in the same individual [31], thus demonstrating that the contralateral ACL is a suitable substitute for the torn ACL for diameter or size measurements.

The ACL diameters computed by us are within the range of previously published data (0.83 ± 0.146 cm) [12]. The ACL injury mechanisms that our study depended on were self-reported. Our study did not take into account the levels of physical activity of the subjects, which may alter the tensile and other mechanical properties of the ACL [10, 32]. It is possible that professional sportspersons may have stronger and firmer ligaments than recreational sportspersons or nonathletes because of recurrent loading of the ligaments [10]. However, to our knowledge, no published data is available to support this hypothesis [10]. Our study assumes matching past physical activity level between control and ACL-injured subjects [10]. Our study also assumes matching ACL injury risk due to extrinsic and intrinsic factors other than ACL diameter between control and ACL-injured subjects [10]. In the future, it is possible that the subjects from the control group may also suffer an injury of the ACL [10]. We tried to control this variable to a significant extent by matching the ages of these two groups; however, disparity in the levels of physical activity affecting both past and future likelihood of an ACL injury remains a shortcoming [10].

## 5. Conclusion

This is the largest study till date to have evaluated normal ACLs by ultrasound. It is also the only one till date to investigate the relationship between ultrasound measured ACL diameter and ACL injury. Ultrasound can be an ideal screening tool in detecting athletes at increased risk of ACL injury. Nevertheless, additional research studies are needed to validate the differences that we observed between control and ACL-injured subjects. ACL diameter (size) may be a predisposing factor for noncontact ACL injury, in addition to various other previously identified extrinsic and intrinsic predisposing factors. An exhaustive study using screening criteria that includes as many of these factors as possible, besides ACL diameter, may turn out to be the most successful in recognizing subjects prone to ligament injuries.

## Figures and Tables

**Table 1:** Demographic, anthropometric, and clinical characteristics of cases and controls.

	ACL injured (*N* = 25)	Control (*N* = 25)	*P* value^*^
Age (years)	33.52 ± 12.22	33.92 ± 7.64	0.890
Height (cm)	170.7 ± 6.37	171.2 ± 5.53	0.768
Weight (kg)	78.68 ± 12.50	80.16 ± 13.15	0.685
BMI	26.98 ± 3.77	27.45 ± 5.10	0.711
Gender			
Male	24 (96%)	24 (96%)	1.00
Female	1 (4%)	1 (4%)	
Ethnicity			
Arab	18 (72%)	18 (72%)	1.00
Non-Arab Asian	6 (24%)	6 (24%)	
Caucasian	1 (4%)	1 (4%)	
ACL injury			
No injury	—	25 (100%)	—
Noncontact injury	25 (100%)	—	
Type of ACL injury			
Complete thickness	17 (68%)	—	—
Partial thickness	8 (32%)	—	
ACL injury side			
Right	10 (40%)	—	—
Left	15 (60%)	—	

^*^ *P* value computed using Chi-square and unpaired *t*-test.

Quantitative variable values were presented in mean ± SD.

**Table 2:** ACL diameter measurements between injured and control subjects.

	ACL injured (mean ± SD) (*N* = 25)	Control (mean ± SD) (*N* = 25)	Mean difference (95% CI)	*P* value^*^
Ultrasound ACL diameter (cm) measurements by radiologist 1	0.61 ± 0.06	0.80 ± 0.07	−0.19 (−0.23 to −0.16)	<0.0001

Ultrasound ACL diameter (cm) measurements by radiologist 2	0.62 ± 0.09	0.81 ± 0.07	−0.18 (−0.23 to −0.14)	<0.0001

Mean ultrasound ACL diameter (cm) measurements by both radiologists	0.62 ± 0.07	0.81 ± 0.06	−0.19 (−0.23 to −0.15)	<0.0001

ACL diameter (MRI versus US) (cm) measurements in 10 controls by radiologist 1		0.84 ± 0.05 versus 0.80 ± 0.06	−0.04 (−0.07 to −0.00)	0.06^†^

MRI ACL diameter (MRI versus US) (cm) measurements in 10 controls by radiologist 2		0.81 ± 0.06 versus 0.79 ± 0.08	−0.19 (−0.06 to −0.03)	0.449^†^

CI: confidence interval.

^*^ *P* value computed using unpaired *t*-test.

^†^Paired *t*-test.

## References

[1] Ferretti A., Conteduca F., de Carli A., Fontana M., Mariani P. P. (1991). Osteoarthritis of the knee after ACL reconstruction. *International Orthopaedics*.

[2] Lohmander L. S., Östenberg A., Englund M., Roos H. (2004). High prevalence of knee osteoarthritis, pain, and functional limitations in female soccer players twelve years after anterior cruciate ligament injury. *Arthritis & Rheumatism*.

[3] Noyes F. R., Mooar P. A., Matthews D. S., Butler D. L. (1983). The symptomatic anterior cruciate-deficient knee. Part I: the long-term functional disability in athletically active individuals. *The Journal of Bone and Joint Surgery*.

[4] Gwinn D. E., Wilckens J. H., McDevitt E. R., Ross G., Kao T.-C. (2000). The relative incidence of anterior cruciate ligament injury in men and women at the United States Naval Academy. *American Journal of Sports Medicine*.

[5] Chandrashekar N., Mansouri H., Slauterbeck J., Hashemi J. (2006). Sex-based differences in the tensile properties of the human anterior cruciate ligament. *Journal of Biomechanics*.

[6] Hashemi J., Chandrashekar N., Mansouri H., Slauterbeck J. R., Hardy D. M. (2008). The human anterior cruciate ligament: sex differences in ultrastructure and correlation with biomechanical properties. *Journal of Orthopaedic Research*.

[7] Davis T. J., Shelbourne K. D., Klootwyk T. E. (1999). Correlation of the intercondylar notch width of the femur to the width of the anterior and posterior cruciate ligaments. *Knee Surgery, Sports Traumatology, Arthroscopy*.

[8] Dienst M., Schneider G., Altmeyer K. (2007). Correlation of intercondylar notch cross sections to the ACL size: a high resolution MR tomographic in vivo analysis. *Archives of Orthopaedic and Trauma Surgery*.

[9] Butler D. L., Kay M. D., Stouffer D. C. (1986). Comparison of material properties in fascicle-bone units from human patellar tendon and knee ligaments. *Journal of Biomechanics*.

[10] Chaudhari A. M. W., Zelman E. A., Flanigan D. C., Kaeding C. C., Nagaraja H. N. (2009). Anterior cruciate ligament-injured subjects have smaller anterior cruciate ligaments than matched controls: a magnetic resonance imaging study. *The American Journal of Sports Medicine*.

[11] Al Moosawi N. M., Mahajan P. S., Al Nahedh Y. S. (2010). MRI evaluation of femoral intercondylar notch width index in cases with and without anterior cruciate ligament injuries. A retrospective study. *Kuwait Medical Journal*.

[12] Chen P.-T., Wu C.-H., Yu C.-W. (2013). Sonography of the normal anterior cruciate ligament: a preliminary report. *Journal of Medical Ultrasound*.

[13] Skovgaard Larsen L. P., Rasmussen O. S. (2000). Diagnosis of acute rupture of the anterior cruciate ligament of the knee by sonography. *European Journal of Ultrasound*.

[14] Suzuki S., Kasahara K., Futami T., Iwasaki R., Ueo T., Yamamuro T. (1991). Ultrasound diagnosis of pathology of the anterior and posterior cruciate ligaments of the knee joint. *Archives of Orthopaedic and Trauma Surgery*.

[15] Ptasznik R., Feller J., Bartlett J., Fitt G., Mitchell A., Hennessy O. (1995). The value of sonography in the diagnosis of traumatic rupture of the anterior cruciate ligament of the knee. *The American Journal of Roentgenology*.

[16] Tate C. M., Williams G. N., Barrance P. J., Buchanan T. S. (2006). Lower extremity muscle morphology in young athletes: an MRI-based analysis. *Medicine and Science in Sports and Exercise*.

[17] Simon R. A., Everhart J. S., Nagaraja H. N., Chaudhari A. M. (2010). A case-control study of anterior cruciate ligament volume, tibial plateau slopes and intercondylar notch dimensions in ACL-injured knees. *Journal of Biomechanics*.

[18] Chandrashekar N., Slauterbeck J., Hashemi J. (2005). Sex-based differences in the anthropometric characteristics of the anterior cruciate ligament and its relation to intercondylar notch geometry: a cadaveric study. *American Journal of Sports Medicine*.

[19] Hewett T. E., Myer G. D., Ford K. R. (2006). Anterior cruciate ligament injuries in female athletes: part 1, mechanisms and risk factors. *The American Journal of Sports Medicine*.

[20] Huston L. J., Greenfield M. L., Wojtys E. M. (2000). Anterior cruciate ligament injuries in the female athlete: potential risk factors. *Clinical Orthopaedics and Related Research*.

[21] Davis I., Ireland M. L., Hanaki S. (2007). ACL injuries: the gender bias. *Journal of Orthopaedic and Sports Physical Therapy*.

[22] Charlton W. P. H., St. John T. A., Ciccotti M. G., Harrison N., Schweitzer M. (2002). Differences in femoral notch anatomy between men and women: a magnetic resonance imaging study. *The American Journal of Sports Medicine*.

[23] Hewett T. E., Lindenfeld T. N., Riccobene J. V., Noyes F. R. (1999). The effect of neuromuscular training on the incidence of knee injury in female athletes: a prospective study. *The American Journal of Sports Medicine*.

[24] Mandelbaum B. R., Silvers H. J., Watanabe D. S. (2005). Effectiveness of a neuromuscular and proprioceptive training program in preventing anterior cruciate ligament injuries in female athletes: 2-Year follow-up. *The American Journal of Sports Medicine*.

[25] Besier T. F., Lloyd D. G., Cochrane J. L., Ackland T. R. (2001). External loading of the knee joint during running and cutting maneuvers. *Medicine and Science in Sports and Exercise*.

[26] Chaudhari A. M. W., Lindenfeld T. N., Andriacchi T. P. (2007). Knee and hip loading patterns at different phases in the menstrual cycle: implications for the gender difference in anterior cruciate ligament injury rates. *The American Journal of Sports Medicine*.

[27] Cowling E. J., Steele J. R. (2001). Is lower limb muscle synchrony during landing affected by gender? Implications for variations in ACL injury rates. *Journal of Electromyography & Kinesiology*.

[28] Hewett T. E., Myer G. D., Ford K. R. (2005). Biomechanical measures of neuromuscular control and valgus loading of the knee predict anterior cruciate ligament injury risk in female athletes: a prospective study. *The American Journal of Sports Medicine*.

[29] Holm I., Fosdahl M. A., Friis A., Risberg M. A., Myklebust G., Steen H. (2004). Effect of neuromuscular training on proprioception, balance, muscle strength, and lower limb function in female team handball players. *Clinical Journal of Sport Medicine*.

[30] Knobloch K., Martin-Schmitt S., Gösling T., Jagodzinski M., Zeichen J., Krettek C. (2005). Prospective proprioceptive and coordinative training for injury prevention in elite female soccer. *Sportverletzung-Sportschaden*.

[31] Jamison S. T., Flanigan D. C., Nagaraja H. N., Chaudhari A. M. (2009). Side-to-side differences in anterior cruciate ligament volume in healthy subjects. *Transactions of the Orthopaedic Research Society*.

[32] Yasuda K., Hayashi K. (1999). Changes in biomechanical properties of tendons and ligaments from joint disuse. *Osteoarthritis and Cartilage*.

